# Bereavement in childhood and young adulthood and the risk of atrial fibrillation: a population-based cohort study from Denmark and Sweden

**DOI:** 10.1186/s12916-022-02707-4

**Published:** 2023-01-05

**Authors:** Hua Chen, Imre Janszky, Mikael Rostila, Dang Wei, Fen Yang, Jiong Li, Krisztina D. László

**Affiliations:** 1 Department of Global Public Health, Karolinska Institutet, Tomtebodavägen 18A, 171 77 Stockholm, Sweden; 2 Department of Public Health and Nursing, Faculty of Medicine, Norwegian University of Science and Technology, Trondheim, Norway; 3 Department of Public Health Sciences, Stockholm University, Stockholm, Sweden; 4 Centre for Health Equity Studies, Stockholm University/Karolinska Institutet, Stockholm, Sweden; 5 Department of Clinical Medicine - Department of Clinical Epidemiology, Aarhus University, Aarhus, Denmark; 6 Department of Public Health and Caring Sciences, Uppsala University, Uppsala, Sweden

**Keywords:** Bereavement, Death of the parent, Death of the sibling, Atrial fibrillation, Stress

## Abstract

**Background:**

Adverse childhood life events are associated with increased risks of hypertension, ischemic heart disease, and stroke later in life. Limited evidence also suggests that stress in adulthood may increase the risk of atrial fibrillation (AF). Whether childhood adversity may lead to the development of AF is unknown. We investigated whether the loss of a parent or sibling in childhood is associated with an increased risk of AF and compared this effect to that of similar losses in young adulthood.

**Methods:**

We studied 6,394,975 live-born individuals included in the Danish (1973–2018) and Swedish Medical Birth Registers (1973–2014). We linked data from several national registers to obtain information on the death of parents and siblings and on personal and familial sociodemographic and health-related factors. We analyzed the association between bereavement and AF using Poisson regression.

**Results:**

Loss of a parent or sibling was associated with an increased AF risk both when the loss occurred in childhood and in adulthood; the adjusted incident rate ratios and 95% confidence intervals were 1.24 (1.14–1.35) and 1.24 (1.16–1.33), respectively. Bereavement in childhood was associated with AF only if losses were due to cardiovascular diseases or other natural causes, while loss in adulthood was associated with AF not only in case of natural deaths, but also unnatural deaths. The associations did not differ substantially according to age at loss and whether the deceased was a parent or a sibling.

**Conclusions:**

Bereavement both in childhood and in adulthood was associated with an increased AF risk.

**Supplementary Information:**

The online version contains supplementary material available at 10.1186/s12916-022-02707-4.

## Background

Atrial fibrillation (AF) is the most common sustained cardiac arrhythmia, with an estimated prevalence of 33 million cases and an annual incidence of 5 million cases globally [1]. Atrial fibrillation is associated with increased risks of mortality and cardiovascular diseases (CVD), including stroke, acute myocardial infarction, and heart failure [2]. A substantial proportion of AF cases may not be attributable to its main known risk factors, i.e., genetic predisposition, advanced age, diabetes mellitus, hypertension, obstructive sleep apnea, obesity, and unhealthy lifestyle [3, 4], emphasizing the importance of identifying novel risk factors [5]. Furthermore, given its strong association with age, AF is often regarded as a disease of the elderly. However, AF occurs—even if with a low incidence—also in childhood and in early adulthood; nevertheless, knowledge about the etiology of the disease in the young is very limited.

Several studies reported that adverse psychological factors in adulthood—primarily work stress [6], adverse life events [7], or emotional distress [8, 9]—are associated with a modestly increased risk of AF. However, evidence in this field is limited and inconsistent as several studies did not find such associations [10, 11]. A recent meta-analysis suggests a modest association between stress assessed in adulthood and the risk of ischemic heart disease and stroke (1.1- to 1.6-fold increased risk) but a higher relative risk in case of stress in childhood (an approximately 2-fold increased risk) [12]. To our knowledge, no previous study investigated whether stress in childhood may increase the risk of AF.

The death of a parent in childhood is one of the most stressful events that a child can experience [13] and is likely to adversely affect the child’s daily routines, psychological functioning, social support, financial situation, and living environment and to lead to attachment difficulties to a partner, lower educational attainment, and unemployment in adulthood [14, 15]. The relationship with the sibling may be the longest and one of the closest over life; thus, loss of a sibling may be a further devastating event in childhood [16]. The acute or chronic stress related to bereavement may lead to changes in the hypothalamic-pituitary-adrenal axis and the autonomic nervous, endocrine, immune, and cardiovascular systems [17, 18], all of which may initiate and/or maintain AF by inducing electrical and structural remodeling of the heart [2, 18–23]. Thus, if stress in childhood is implicated in the etiology of AF, we may expect to observe an association between bereavement in childhood and AF risk.

In this prospective study based on a bi-national Nordic cohort, we aimed to investigate whether severe stress, defined as the death of a parent or sibling, in childhood is associated with an increased AF risk. Secondary aims were (1) to analyze whether the association differs according to the relationship to the deceased, the relative’s cause of death, and age at loss in childhood and (2) to estimate similar associations for loss in adulthood and to compare them to those observed in case of childhood loss, given the limited and inconsistent evidence regarding the role of stress in adulthood in the etiology of AF.

## Methods

### Study population and design

Our study population included live-born individuals recorded in the Danish Medical Birth Register (DMBR) during 1973–2018 and in the Swedish Medical Birth Register (SMBR) during 1973–2014 (*n* = 7,150,339). We linked the offsprings to parents, siblings, grandparents, and parents’ siblings by means of the Danish MBR and Civil Registration System, and the Swedish MBR and Multi-Generation Register using the unique personal identification number assigned to residents in both countries. We linked the MBRs with further nationwide population-based registers to obtain information on health-related, demographic, and socioeconomic factors (Additional file 1: Table S1). We excluded offsprings with missing information on the father in our data (*n* = 755,364), resulting in 6,394,975 offsprings, 2,850,011 from the DMBR, and 3,544,964 from the SMBR being included in the final analyses.

### Exposure

We defined exposure as the first death of a parent before (applicable only to fathers) or after birth or the first death of a sibling after birth. We obtained information on these relatives’ date and cause of death from the Danish Civil Registration System and from the Swedish Cause of Death Register. We classified the exposure according to (1) the offspring’s age at loss (≤ 18 or > 18 years and further as ≤ 5, 6–12, 13–18, 19–25, 26–30, or > 30 years); (2) the parent’s or the sibling’s cause of death (due to CVD, other natural causes, or unnatural causes using the International Classification of Diseases (ICD) codes presented in Additional file 1: Table S2); and (3) the relationship to the deceased (parent or sibling).

### Outcome

We retrieved information on AF from the National Hospital Register in Denmark and from the Patient Register in Sweden; we searched both the primary and the secondary diagnoses for the ICD codes shown in Additional file 1: Table S2. The positive predictive value of the diagnosis of AF is high in both the Danish National Hospital Register and Swedish Patient Register (99% and 96%, respectively) [24, 25]. We followed offsprings from birth until the first diagnosis of AF, death, emigration, or end of follow-up (December 31, 2018, in Denmark and December 31, 2014, in Sweden), whichever came first.

### Covariates

We obtained information on the offspring’s sex and date of birth, maternal age at offspring’s birth, and the parents’ country of origin from the Danish Civil Registration System, the DMBR, the Swedish Total Population Register, and the SMBR, respectively. We obtained information on the lifetime highest educational level of the offspring and the parents and on maternal and paternal income from the Integrated Database for Labor Market Research in Denmark and from the Education Register and the Register of Incomes and Taxes in Sweden. To define education, we used information on both parents’ highest educational level; in case information for one parent was missing, we used information available for the other parent only. We considered data on maternal and paternal income from the offspring’s birth year; in case such information was not available for the birth year, we used information from the closest available year, up to 5 years before or after the birth year. Maternal height and weight in early pregnancy, smoking, and gestational age were extracted from the DMBR and from the SMBR. We calculated maternal body mass index (BMI) by dividing weight in kilograms by the square of height in meters. We obtained information on maternal hypertension and diabetes and parents’ history of CVD before the offspring’s birth from the Danish National Hospital Register and from the SMBR and the Patient Register, respectively, in Sweden. We obtained information on family (grandparents and biological uncles/aunts) history of CVD before the offspring’s birth from the National Hospital Register and the Civil Registration System in Denmark and from the Patient Register and the Cause of Death Register in Sweden. We obtained information on offspring’s psychiatric disorders during follow-up and on parents’ and family history of psychiatric disorders before the offspring’s birth from the National Hospital Register and the Psychiatric Central Register in Denmark and the Patient Register in Sweden. We obtained information on heart failure, acute myocardial infarction, hypertension, and diabetes in the offspring during follow-up from the National Hospital Register in Denmark and the Patient Register in Sweden. We present the ICD codes used to retrieve medical conditions in Additional file 1: Table S2 and the categorization of each covariate in Table 1.

**Table 1:** Characteristics of offsprings according to a bereavement in childhood and in adulthood

Variables	Unexposed (***n*** = 5,771,095), ***N*** (%)	Exposure type	
		Any loss in childhood (***n*** = 248,970), ***N*** (%)	Any loss in adulthood (***n*** = 374,910), ***N*** (%)
Country			
Denmark	2,533,112 (43.9)	120,338 (48.3)	196,561 (52.4)
Sweden	3,237,983 (56.1)	128,632 (51.7)	178,349 (47.6)
Sex			
Men	2,955,678 (51.2)	127,468 (51.2)	193,714 (51.7)
Women	2,811,917 (48.7)	121,353 (48.7)	181,192 (48.3)
Missing	3500 (0.1)	149 (0.1)	4 (< 0.1)
Gestational age of offspring (weeks)			
< 32	43,800 (0.8)	3657 (1.5)	1642 (0.4)
32–36	272,138 (4.7)	14,202 (5.7)	14,597 (3.9)
> 36	5,152,821 (89.3)	202,153 (81.2)	258,182 (68.9)
Missing	302,336 (5.2)	28,958 (11.6)	100,489 (26.8)
Parents’ country of birth^a^			
Denmark or Sweden	4,941,683 (85.6)	202,582 (81.4)	334,062 (89.1)
Other countries	829,412 (14.4)	46,388 (18.6)	40,848 (10.9)
Parents’ highest educational level (years)^b^			
0–9	342,608 (5.9)	42,217 (17.0)	65,840 (17.6)
10–14	3,144,996 (54.5)	145,202 (58.3)	224,346 (59.8)
≥ 15	2,262,151 (39.2)	60,435 (24.3)	84,192 (22.5)
Missing	21,340 (0.4)	1116 (0.4)	432 (0.1)
Maternal income at offspring’s birth^c^			
Low tertile	1,812,644 (31.4)	89,081 (35.8)	121,605 (32.4)
Middle tertile	1,945,845 (33.7)	73,090 (29.4)	106,877 (28.5)
High tertile	1,917,486 (33.2)	76,422 (30.7)	105,445 (28.1)
Missing	95,120 (1.6)	10,377 (4.2)	40,983 (10.9)
Paternal income			
Low tertile	1,857,048 (32.2)	102,063 (41.0)	120,052 (32.0)
Middle tertile	1,902,257 (33.0)	69,308 (27.8)	106,460 (28.4)
High tertile	1,903,458 (33.0)	66,366 (26.7)	107,096 (28.6)
Missing	108,332 (1.9)	11,233 (4.5)	41,302 (11.0)
Maternal age at offspring’s birth (years)			
< 20	131,853 (2.3)	9215 (3.7)	15,162 (4.0)
20–24	1,059,666 (18.4)	49,745 (20.0)	87,263 (23.3)
25–29	2,061,402 (35.7)	76,435 (30.7)	129,050 (34.4)
30–34	1,706,237 (29.6)	66,883 (26.9)	93,727 (25.0)
> 34	811,937 (14.1)	46,692 (18.8)	49,708 (13.3)
Maternal smoking in early pregnancy			
No	3,373,378 (58.5)	77,711 (31.2)	33,144 (8.8)
Yes	682,436 (11.8)	43,983 (17.7)	23,252 (6.2)
Missing	1,715,281 (29.7)	127,276 (51.1)	318,514 (85.0)
Maternal BMI in early pregnancy (kg/m^2^)			
< 30	2,567,603 (44.5)	57,396 (23.1)	29,859 (8.0)
≥ 30	292,351 (5.1)	6458 (2.6)	1481 (0.4)
Missing	2,911,141 (50.4)	185,116 (74.4)	343,570 (91.6)
Maternal hypertension before offspring’s birth			
No	5,321,343 (92.2)	218,775 (87.9)	289,602 (77.2)
Yes	266,451 (4.6)	10,703 (4.3)	9741 (2.6)
Missing	183,301 (3.2)	19,492 (7.8)	75,567 (20.2)
Maternal diabetes before offspring’s birth			
No	5,513,046 (95.5)	227,012 (91.2)	297,769 (79.4)
Yes	74,748 (1.3)	2466 (1.0)	1574 (0.4)
Missing	183,301 (3.2)	19,492 (7.8)	75,567 (20.2)
Parents’ CVD before offspring’s birth			
No	5,276,302 (91.4)	215,185 (86.4)	290,662 (77.5)
Yes	311,492 (5.4)	14,293 (5.7)	8681 (2.3)
Missing	183,301 (3.2)	19,492 (7.8)	75,567 (20.2)
Parents’ psychiatric disorders before offspring’s birth			
No	5,236,886 (90.9)	220,964 (88.8)	360,603 (96.2)
Yes	524,209 (9.1)	28,006 (11.2)	14,307 (3.8)
Family history of CVD before offspring’s birth^d^			
No	2,273,064 (39.4)	62,183 (25.0)	95,224 (25.4)
Yes	2,460,022 (42.6)	95,199 (38.2)	92,884 (24.8)
Missing	1,038,009 (18.0)	91,588 (36.8)	186,802 (49.8)
Family history of psychiatric disorders before offspring’s birth^d^			
No	3,306,066 (57.3)	96,215 (38.6)	135,857 (36.2)
Yes	1,296,122 (22.5)	50,782 (20.4)	39,885 (10.6)
Missing	1,168,907 (20.3)	101,973 (41.0)	199,168 (53.1)

*BMI* body mass index, *CVD* cardiovascular diseases

^a^Classified according to whether both parents were from the studied countries (Denmark or Sweden)

^b^Defined as the highest education of the two parents; if information on education was missing for one of the parents, we used information only from the other parent

^c^Classified based on the tertile distribution of each calendar year

^d^A family history of CVD and psychiatric disorders was defined based on a record of each of these disorders in the offsprings’ grandparents and uncles/aunts

### Statistical analyses

We used Poisson regression models to estimate the association between the death of a parent or sibling and the risk of AF. We treated exposure as a time-varying variable, i.e., exposed offsprings contributed person-time from birth until the death of the parent or sibling to the unexposed group and to the exposed group afterwards; unexposed offsprings contributed person-time only to the unexposed group. We ran analyses with any loss in childhood and in adulthood, as well as with these exposures further categorized according to the cause of the parent’s or sibling’s death and the child’s age at loss (≤ 5, 6–12, 13–18, 19–25, 26–30, or > 30 years) and separately for the loss of a parent (and for mother and father) and loss of a sibling. We ran models adjusted for time since birth and calendar year of follow-up and models additionally adjusted for country, maternal age at the offspring’s birth, and the parents’ country of origin, highest education, and history of psychiatric disorders; we treated time since birth (in 5-year intervals) and calendar year of follow-up (1973–1979, in 10-year intervals from 1980) as time-varying variables.

We investigated whether the risk of AF varies according to the time since the loss by splitting the follow-up of the exposed as < 3 months, 3 months to 1 year, 1–5 years, 5–10 years, and > 10 years. Given that information on several covariates (e.g., gestational age, maternal and paternal income, maternal smoking and BMI in early pregnancy, maternal hypertension and diabetes, parents’ history of CVD, and family history of CVD and psychiatric disorders before the offspring’s birth) was available only for a sub-cohort (mainly because such information was recorded in the registers in the later parts of our study period) (Additional file 1: Table S1), we adjusted for these potential confounders among those with such data. We adjusted for the offspring’s highest educational level in addition to the factors in the main model to study whether this variable mediated the association between parent’s death in childhood and the risk of AF. To investigate whether offspring’s heart failure, acute myocardial infarction, hypertension, diabetes, and psychiatric disorders contributed to the association studied, we classified exposed individuals according to whether they had these medical conditions after loss. We run analyses stratified by sex, study country, and parents’ highest education to analyze whether the association of interest differed according to these characteristics. We ran analyses after excluding offsprings (1) whose father died before birth (*n* = 1762) and (2) whose parent or sibling died before age 2 given concerns that these losses might eventually not cause stress in these very young offsprings (*n* = 32,315).

We used SAS 9.4. (SAS Institute Inc., Cary, NC, USA) for the analyses.

## Results

Of the 6,394,975 offsprings, 248,970 (3.9%) were exposed to a parent’s or sibling’s death in childhood and 374,910 (5.9%) in adulthood. The characteristics of offsprings by exposure status are presented in Table 1.

**Fig. 1:**
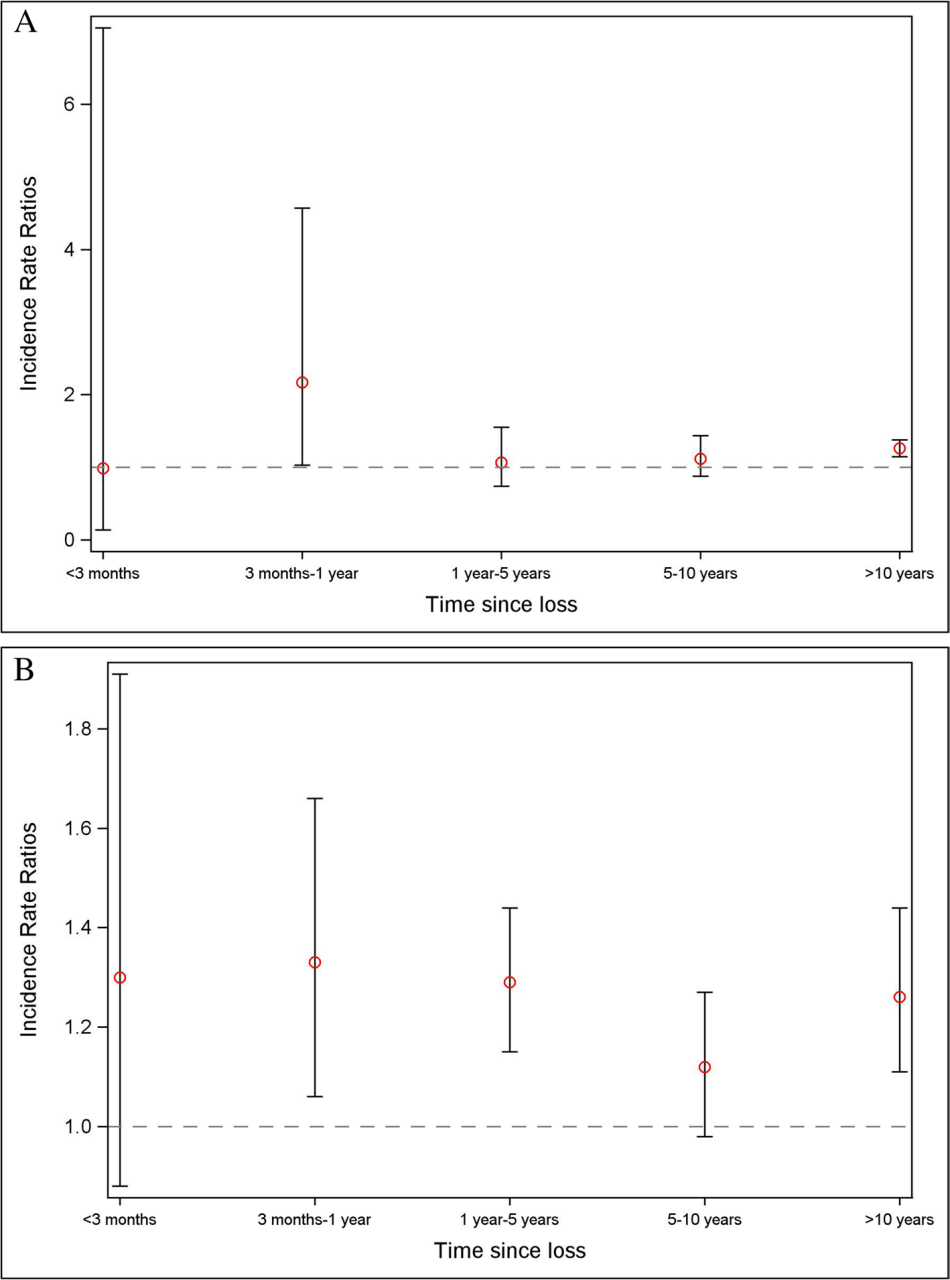
Adjusted incidence rate ratios for atrial fibrillation by time since the loss. **A** Death of a parent or sibling in childhood. **B** Death of a parent or sibling in adulthood. We adjusted for time since birth, calendar year, country, maternal age at the offspring’s birth, and the parents’ country of origin, highest education, and history of psychiatric disorders

A total of 8723 (6.5/10^5^ person-years) offsprings had AF during follow-up. The median age at the time of the AF was 29 years (interquartile range: 22–34) in the overall cohort. The median age at the time of AF was 27 years (interquartile range: 21–33) among the unexposed offsprings, 30 years (interquartile range: 23–35) among offsprings exposed to the death of a parent or sibling in childhood, and 35 years (interquartile range: 31–39) among offsprings exposed to loss in adulthood. Altogether, 881 (10.1% of the total AF cases) individuals were diagnosed with AF before the age of 18 years. Loss of a parent or sibling in childhood was associated with an increased risk of AF; the adjusted incidence rate ratio (IRR) and 95% confidence intervals (CI) for loss in childhood were 1.24 (1.14–1.35) (Table 2). Bereavement in childhood was associated with an increased risk of AF only if losses were due to CVD or other natural causes. The association was largely similar across all the categories of age at loss in childhood and was similar in the case of the death of a parent and sibling. The association was observed for paternal death in childhood but not for maternal death. The association observed in childhood was similar to that for the loss of a parent or sibling in adulthood, in case of which the IRR (95% CI) was 1.24 (1.16–1.33) (Table 3). However, losses in adulthood were associated with AF not only in cases of natural deaths, but also in cases of unnatural deaths of relatives. The association did not substantially differ when we more finely classified exposure according to the age of the offspring at loss in adulthood. The risk of AF was highest during the 3 months to 1 year after loss, both for losses in childhood and in adulthood (Fig. 1).

**Table 2:** Adjusted incidence rate ratios for atrial fibrillation according to exposure to a bereavement in childhood

Exposure	Death of a parent or a sibling			Death of a parent		Death of a sibling^**a**^	
	Events/person-years	Model 1 IRR (95% CI)^**b**^	Model 2 IRR (95% CI)^**c**^	Events/person-years	Model 2 IRR (95% CI)^**c**^	Events/person-years	Model 2 IRR (95% CI)^**c**^
Unexposed	7158/127,901,121	1.00	1.00	7356/129,284,682	1.00	7919/126,530,901	1.00
Any loss in childhood	615/4,409,805	1.26 (1.16–1.37)	1.24 (1.14–1.35)	473/3,210,311	1.25 (1.14–1.37)	158/1,279,249	1.18 (1.01–1.38)
Cause of death^d^							
Death due to CVD	143/604,057	1.96 (1.66–2.32)	1.93 (1.64–2.28)	141/581,394	1.95 (1.65–2.31)	6/33,493	1.62 (0.73–3.62)
Other natural deaths	310/2,349,870	1.19 (1.07–1.34)	1.17 (1.05–1.32)	218/1,574,633	1.14 (0.99–1.30)	97/808,789	1.21 (0.99–1.48)
Unnatural death	158/1,435,306	1.03 (0.88–1.21)	1.02 (0.87–1.19)	111/1,040,552	0.98 (0.81–1.19)	54/429,692	1.09 (0.84–1.43)
Age of offspring at the time of loss (in years)							
0–5	139/1,451,840	1.24 (1.05–1.47)	1.22 (1.03–1.44)	77/840,906	1.16 (0.92–1.46)	62/619,952	1.23 (0.96–1.58)
6–12	221/1,596,481	1.31 (1.14–1.50)	1.29 (1.12–1.47)	173/1,210,311	1.31 (1.13–1.53)	55/415,036	1.21 (0.93–1.58)
13–18	255/1,361,484	1.24 (1.09–1.40)	1.22 (1.07–1.38)	223/1,159,094	1.24 (1.08–1.41)	41/244,262	1.07 (0.79–1.46)
Sex of the deceased parent							
Mother	NA	NA	NA	110/967,799	0.94 (0.78–1.14)	NA	NA
Father	NA	NA	NA	363/2,242,512	1.39 (1.24–1.54)	NA	NA

*IRR* incidence rate ratio, *CI* confidence intervals, *CVD* cardiovascular diseases

^a^Offsprings without any sibling at birth were excluded

^b^Adjusted for time since birth and calendar year

^c^Adjusted for time since birth, calendar year, country, maternal age at offspring’s birth, and the parents’ country of origin, highest education, and history of psychiatric disorders

^d^Offsprings with missing data on this type of exposure were excluded

**Table 3:** Adjusted incidence rate ratios for atrial fibrillation according to exposure to a bereavement in adulthood

Exposure	Death of a parent or a sibling			Death of a parent		Death of a sibling^**a**^	
	Events/person-years	Model 1 IRR (95% CI)^**b**^	Model 2 IRR (95% CI)^**c**^	Events/person-years	Model 2 IRR (95% CI)^**c**^	Events/person-years	Model 2 IRR (95% CI)^**c**^
Unexposed	7158/127,901,121	1.00	1.00	7356/129,284,682	1.00	7919/126,530,901	1.00
Any loss in adulthood	950/2,801,341	1.26 (1.17–1.35)	1.24 (1.16–1.33)	894/2,617,274	1.23 (1.14–1.32)	116/346,867	1.22 (1.01–1.46)
Cause of death^d^							
Death due to CVD	244/588,453	1.54 (1.35–1.75)	1.52 (1.34–1.74)	243/589,926	1.50 (1.32–1.71)	17/29,595	1.93 (1.20–3.11)
Other natural deaths	557/1,791,782	1.17 (1.07–1.28)	1.15 (1.06–1.26)	568/1,748,276	1.15 (1.05–1.26)	39/135,859	1.00 (0.73–1.36)
Unnatural death	122/400,240	1.25 (1.04–1.50)	1.24 (1.03–1.48)	78/260,991	1.20 (0.96–1.50)	57/177,268	1.23 (0.95–1.60)
Age of offspring at the time of loss (in years)							
19–25	389/1,406,237	1.30 (1.17–1.44)	1.28 (1.15–1.42)	349/1,280,672	1.25 (1.12–1.39)	50/188,884	1.21 (0.92–1.61)
26–30	252/739,687	1.21 (1.06–1.37)	1.19 (1.05–1.35)	243/699,174	1.20 (1.05–1.37)	30/85,628	1.19 (0.83–1.70)
> 30	309/655,418	1.25 (1.11–1.42)	1.24 (1.10–1.39)	302/637,428	1.23 (1.09–1.39)	36/72,355	1.24 (0.89–1.73)
Sex of the deceased parent							
Mother	NA	NA	NA	296/856,798	1.24 (1.10–1.40)	NA	NA
Father	NA	NA	NA	598/1,760,476	1.22 (1.12–1.33)	NA	NA

*IRR* incidence rate ratio, *CI* confidence intervals, *CVD* cardiovascular diseases

^a^Offsprings without any sibling at birth were excluded

^b^Adjusted for time since birth and calendar year

^c^Adjusted for time since birth, calendar year, country, maternal age at offspring’s birth, and the parents’ country of origin, highest education, and history of psychiatric disorders

^d^Offsprings with missing data on this type of exposure were excluded

The association between a parent’s or a sibling’s death in childhood and adulthood and the risk of AF did not substantially alter after additional adjustment for gestational age, maternal and paternal income, maternal smoking and BMI in early pregnancy, maternal hypertension and diabetes, parents’ history of CVD, and a family history of CVD and psychiatric disorders before the offspring’s birth (Additional file 1: Table S3). Similarly, adjustment for the offspring’s highest educational level did not substantially change the association between the parent’s death in childhood and the risk of AF; the corresponding adjusted IRRs (95% CI) before and after adjusting for education were 1.25 (1.14–1.38) and 1.22 (1.10–1.34), respectively. The IRR for AF was higher among the exposed individuals with heart failure, acute myocardial infarction, and hypertension after the loss during follow-up than among the exposed individuals without these diseases; there was no similar pattern in the analyses regarding diabetes and psychiatric disorders during the follow-up (Additional file 1: Table S4). The magnitude of the association between bereavement, both in childhood and in adulthood, and the risk of AF was slightly higher among women than among men; there were no differences in the studied associations according to study country or the parents’ highest education (Additional file 1: Table S5). The results were similar to those from the main analyses after we excluded the offsprings who lost the father before birth [IRR (95% CI): 1.26 (1.14–1.34) for childhood loss] and who lost a parent or sibling up to the age of 2 years [IRR (95% CI): 1.26 (1.15–1.37) for childhood loss].

## Discussion

In this large prospective study based on a bi-national cohort, we found that the death of a parent or sibling, both in childhood and in adulthood, was associated with an increased risk of AF. The association was largely similar across all the categories of age at loss and did not differ substantially according to whether the loss concerned a parent or a sibling. Bereavement in childhood was associated with AF only in case of losses due to CVD or other natural causes, while the loss in adulthood was associated with AF in case of unnatural deaths as well.

### Comparison with earlier studies

To our knowledge, this is the first study to investigate the association between childhood adversity and the risk of AF. The findings that the death of a parent or sibling, both in childhood and in early adulthood, was associated with an increased risk of AF corroborate the results of earlier studies showing that individuals who lost a partner or child had higher risks of AF than their unexposed counterparts [5, 26]. Similarly, the results were in line with the findings of several [6–9], though not all [10, 11], previous studies suggesting that job stress, adverse life events, psychological distress, and certain psychiatric disorders are associated with AF, as well as those of several earlier studies reporting an increased risk of cardiovascular mortality or incident ischemic heart diseases, stroke, or heart failure in adulthood after experiencing the death of a parent [15, 27–29] or sibling in childhood [30] or after exposure to other childhood adversities [31]. Our study contributes to the existing literature on the role of stress and adverse life events in the etiology of AF by investigating the exposure to death of a close relative in childhood (overall and by subtypes) in a large bi-national cohort study, by comparing the effects of childhood and adult bereavement, and by considering a large number of potential confounders of this association.

We found that the risk of AF was increased when the loss—in childhood or adulthood—was due to CVD or other natural causes. This finding is in line with that of several other studies reporting the highest risks of acute myocardial infarction, ischemic heart disease, stroke, and cardiovascular mortality after the loss of a parent in childhood [15, 28] and of a sibling in childhood and adulthood [32–34] if the relative’s death was due to natural, in particular, cardiovascular deaths. One potential explanation for these findings is confounding due to shared familial cardiovascular risk factors, e.g., genetic susceptibility, diet, or living environment. Alternatively, it is possible that childhood adversity may increase the risk of AF only among those with a cardiometabolic vulnerability, which is likely to be more pronounced among those who lost a parent due to CVD or other related diseases. In contrast, unnatural deaths of the relatives—which are less likely to be affected by cardiovascular risk factors clustering in the family—were associated with the risk of AF only if the loss occurred in adulthood, providing stronger support for the hypotheses that stress-related mechanisms may operate.

The association between the parent’s or the sibling’s death in childhood and the risk of AF may differ according to the age of the child at loss. The first few years of life are a sensitive period regarding the loss of a parent as the early interaction with caregivers is critical for the development of the brain architecture and the programming of stress reactivity [35]. Adolescence is another potentially sensitive period, given that adolescents exposed to stress may adopt negative coping strategies in terms of adverse health behaviors [36]. Nevertheless, we found no evidence that the risk of AF differed according to the minor child’s age at loss. Similarly, we expected that the death of parents may have a stronger emotional impact and be more closely related to the adverse health outcomes than the death of a sibling in childhood. Similarly, mothers are often the primary attachment figures and are probably more involved in their children’s upbringing than fathers [37, 38]. However, the associations did not differ substantially when comparing the loss of a sibling to that of a parent, and a link between the death of a parent in childhood and the risk of AF was observed only in the case of deaths of the father, but not of the mother. We speculate that a possible explanation for this latter finding may be related to better statistical power in case of paternal deaths in childhood or a higher proportion of cardiovascular and other natural deaths among the fathers than the mothers during childhood.

### Potential linking mechanisms

It is not clear why we did not find an association between bereavement due to unnatural causes in childhood—which are less likely to be affected by cardiovascular risk factors than natural deaths—and the risk of AF. One explanation may be related to the earlier observation in adult samples that the AF risk increase after the loss was more pronounced in the short than in the long term [5]. A similar triggering effect may not apply for pediatric AF, a condition with largely unknown, but potentially different etiology than AF in adulthood, nor for the relation between exposure in childhood and AF in adulthood. Moreover, detecting AF in children may be more difficult than in adults due to the differences in symptom presentation or the lower quality of the information provided by children on their symptoms or because physicians would normally not expect AF in childhood [39]. Though we did not observe an association in the case of unnatural deaths in childhood, it is still possible that stress in childhood may increase the risk of AF if it interacts with cardiometabolic vulnerability.

Bereavement, in childhood or adulthood, may increase the risk of AF through several pathways. An increasing number of studies suggest an elevated autonomic nervous system activity before episodes of paroxysmal AF [40, 41], while others document a higher frequency of stressful life events before paroxysmal AF [42] or first AF diagnoses [5, 26], i.e., a triggering effect. In the longer term, bereavement stress and the associated chronic activation and dysregulation of the hypothalamic-pituitary-adrenal axis and the autonomic nervous system may increase the risk of psychological distress, mental disorders, adverse lifestyle, and unfavorable changes in the autonomic tone and in endocrine, immune, inflammatory, hemodynamic, and cardiovascular activity [17, 18]. These in turn may promote electrical changes and the structural remodeling of the heart [18–22], thereby triggering and sustaining AF [2, 23]. Our findings of a lower AF risk in bereaved individuals who did not develop compared to those who developed ischemic heart diseases, heart failure and hypertension after the loss, may be supportive of these hypotheses, though differences in detection of AF may have also contributed to these findings.

### Strengths and limitations

Our study had several strengths. First, we had prospectively collected information on offsprings from birth up to early middle age for both exposure and outcome. Second, the large sample size yielded us the possibility to detect modest associations, to conduct several important sub-analyses according to the characteristics of bereavement, or to relevant sociodemographic factors. Third, the extensive register linkages allowed us to adjust for several important covariates. Nevertheless, our findings need to be interpreted in light of some limitations. First, though we adjusted for several covariates, we did not have information on other potential confounders such as genetic factors, lifestyle, and living environment. Second, our findings may only be generalized to countries comparable to Denmark and Sweden in terms of their welfare system, sociocultural context, and quality and accessibility of healthcare. We could expect that the association between bereavement and AF would be stronger in countries with more limited resources for bereaved children or where adults rely to a larger extent on support from family than the state. Third, though the positive predictive value of the AF diagnoses in the Danish and Swedish patient register has been shown to be very high [24, 25], we may have missed (1) some mild AF cases and (2) AF diagnoses before the coverage of the Danish Hospital Register and the Swedish Patient Register with respect to specialized outpatient care became complete. Similarly, since information on psychiatric disorders was retrieved from specialized outpatient and inpatient care, we may have missed the milder forms of psychiatric diseases in our variables concerning parents’ and family history of psychiatric disorders. Fourth, as information on cardiovascular medications was available only for individuals born after 2005 in Sweden, our possibilities to investigate the importance of antihypertensive or other medication in the studied association were limited.

## Conclusions

In this large bi-national cohort study, we found an association between death of a parent or sibling and an increased risk of AF. The finding that the association between bereavement in childhood was confined to natural deaths of the relatives suggests that confounding by familial cardiovascular factors or an interaction between familial cardiometabolic risk factors and stress is likely an explanation for this link. In contrast, the finding that losses due to unnatural causes in adulthood were associated with AF risk may be supportive of stress-related mechanisms for the link between bereavement and AF.

## Supplementary Information

**Additional file 1: Table S1.** Registers included in the linkage and retrieved variables. **Table S2.** International Classification of Diseases codes used to classify causes of death and medical conditions. **Table S3.** Adjusted incidence rate ratios for atrial fibrillation by death of a parent or a sibling among offsprings with data on specific covariates. **Table S4.** Adjusted incidence rate ratios for atrial fibrillation according to bereavement in analyses considering potential mediators. **Table S5.** Adjusted incidence rate ratios for the association between death of a parent or a sibling in childhood and adulthood and atrial fibrillation in stratified analyses.

## Data Availability

The data may be obtained from third parties (Statistics Denmark, Statistics Sweden, and the Swedish National Board of Health and Welfare) and are not publicly available.

## Abbreviations

AF: Atrial fibrillation
BMI: Body mass index
CI: Confidence interval
CVD: Cardiovascular diseases
DMBR: Danish Medical Birth Register
ICD: International Classification of Diseases
IRR: Incidence rate ratio
SMBR: Swedish Medical Birth Register
