# Supplementary material for: Bereavement in childhood and young adulthood and the risk of atrial fibrillation: a population-based cohort study from Denmark and Sweden
Source: BMC Med. 2023 Jan 5;21:8. doi: 10.1186/s12916-022-02707-4 (PMC9814172; doi:10.1186/s12916-022-02707-4)
Supplement: Supplementary file 1 — Additional file 1: Table S1. Registers included in the linkage and retrieved variables. Table S2. International Classification of Diseases codes used to classify causes of death and medical conditions. Table S3. Adjusted incidence rate ratios for atrial fibrillation by death of a parent or a sibling among offsprings with data on specific covariates. Table S4. Adjusted incidence rate ratios for atrial fibrillation according to bereavement in analyses considering potential mediators. Table S5. Adjusted incidence rate ratios for the association between death of a parent or a sibling in childhood and adulthood and atrial fibrillation in stratified analyses. [file 12916_2022_2707_MOESM1_ESM.docx]

**Additional file 1 to:**

**Bereavement in childhood and young adulthood and the risk of atrial fibrillation: a population-based cohort study from Denmark and Sweden**

Hua Chen^1*^, Imre Janszky^1,2^, Mikael Rostila^3,4^, Dang Wei^1^, Fen Yang^1^, Jiong Li^5^, Krisztina D. László^1,6*^

^1^Department of Global Public Health, Karolinska Institutet, Stockholm, Sweden

^2^Department of Public Health and Nursing, Faculty of Medicine, Norwegian University of Science and Technology, Trondheim, Norway

^3^Department of Public Health Sciences, Stockholm University, Stockholm, Sweden

^4^Centre for Health Equity Studies, Stockholm University/Karolinska Institutet, Stockholm, Sweden

^5^Department of Clinical Medicine - Department of Clinical Epidemiology, Aarhus University, Aarhus, Denmark

^6^Department of Public Health and Caring Sciences, Uppsala University, Uppsala, Sweden

***Correspondence:** [hua.chen@ki.se](mailto:hua.chen@ki.se) or krisztina.laszlo@ki.se

Department of Global Public Health, Karolinska Institutet, Tomtebodavägen 18A, Stockholm, 171 77, Sweden

**Table S1.** Registers included in the linkage and retrieved variables

| **Registers** | **Retrieved variables** | **Period with available data** |
| --- | --- | --- |
| Denmark |  |  |
| Medical Birth Register | Sex | 1973-2018 |
|  | The offspring’s date of birth | 1973-2018 |
|  | Gestational age | 1978-2018 |
|  | Linkage to mother | 1973-2018 |
|  | Linkage to father | 1991-2016 |
|  | Maternal age at offspring’s birth | 1973-2018 |
|  | Maternal smoking | 1991-2018 |
|  | Maternal weight and height in early pregnancy | 2003-2018 |
| Civil Registration System | Sex | 1973-2018 |
|  | The offspring’s date of birth | 1973-2018 |
|  | The mother’s date of birth | 1968-2018 |
|  | Parents’ country of origin | 1986-2018 |
|  | Date and cause of death | 1970-2018 |
|  | Linkage to parents, grandparents and parents’ siblings | 1968-2018 |
|  | Emigration date | 1973-2018 |
| National Hospital Register^a^ | Date and diagnosis of hospitalization/specialized outpatient care | 1977-2018 |
| Psychiatric Central Register^b^ | Date and diagnosis of psychiatric hospitalizations/outpatient psychiatric care | 1969-2018 |
| The Integrated Database for Longitudinal Labor Market Research | Education | 1980-2018 |
|  | Income | 1980-2015 |
| Sweden |  |  |
| Medical Birth Register | Sex | 1973-2014 |
|  | The offspring’s date of birth | 1973-2014 |
|  | Gestational age | 1973-2014 |
|  | Linkage to the mother | 1973-2014 |
|  | Maternal smoking | 1982-2014 |
|  | Maternal height in early pregnancy | 1982-2014 |
|  | Maternal weight in early pregnancy | 1982-1989, 1992-2014 |
|  | Maternal hypertension and diabetes before or during pregnancy | 1973-2014 |
| Total Population Register | The mother’s year of birth | 1973-2014 |
|  | The parents’ country of origin | 1973-2014 |
|  | Emigration date | 1973-2014 |
| Multi-Generation Register | Linkages to parents, grandparents and parents’ siblings | 1961-2014 |
| Cause of Death Register | Date and cause of death | 1952-2014 |
| Patient Register^c^ | Date and diagnosis of hospitalization/  Specialized outpatient care | 1969-2014 |
| Education Register | Education | 1990-2014 |
| Register of Incomes and Taxes | Personal income | 1972-2014 |

^a^Includes all inpatient diagnoses since 1977 and all outpatient diagnoses since 1995.

^b^Includes data on psychiatric hospitalizations since 1969 and on outpatient psychiatric care since 1995.

^c^Inpatient diagnoses are available since 1969, the coverage of the inpatient care data became nationwide in 1987; data on specialized outpatient care are included from 2001.

**Table S2.** International Classification of Diseases codes used to classify causes of death and medical conditions

| **Medical condition** | **ICD-codes** | | | | |
| --- | --- | --- | --- | --- | --- |
|  | **ICD-6^a^** | **ICD-7^a^** | **ICD-8^a^** | **ICD-9^a^** | **ICD-10^a^** |
| Denmark |  |  |  |  |  |
| Cause of death of the parent or the sibling |  |  |  |  |  |
| Cardiovascular death | NA | NA | 390-458 | NA | I00-I99 |
| Other natural cause | NA | NA | The rest of the codes | NA | The rest of the codes |
| Unnatural death | NA | NA | 7959, 79621, 800-999 | NA | R95, R96, R98, V01-Y98 |
| The outcome of interest |  |  |  |  |  |
| Atrial fibrillation | NA | NA | 427.93, 427.94 | NA | I48 |
| Other variables |  |  |  |  |  |
| Maternal hypertension | NA | NA | 400-404, 63700, 63703, 63704, 63709, 63719 | NA | I10-I15, O10, O11, O13-O16 |
| Maternal diabetes | NA | NA | 249, 250 | NA | E10-E14, O24 |
| Cardiovascular diseases in parents | NA | NA | 390-458 | NA | I00-I99 |
| Psychiatric diseases in parents | NA | NA | 290-315 | NA | F00-F99 |
| Cardiovascular diseases in the family | NA | NA | 390-458 | NA | I00-I99 |
| Psychiatric diseases in the family | NA | NA | 290-315 | NA | F00-F99 |
| Acute myocardial infarction in the offspring | NA | NA | 410 | NA | I21, I22 |
| Heart failure in the offspring |  |  | 42709, 42710, 42711, 42719 |  | I11.0, I13.0, I13.2, I50 |
| Hypertension in the offspring | NA | NA | 400-404, 63700, 63703, 63704, 63709, 63719 | NA | I10-I15, O10, O11, O13-O16 |
| Diabetes in the offspring | NA | NA | 249, 250 | NA | E10-E14, O24 |
| Psychiatric diseases in the offspring | NA | NA | 290-315 | NA | F00-F99 |
| Sweden |  |  |  |  |  |
| Cause of death of the parent or the sibling |  |  |  |  |  |
| Cardiovascular death | NA | NA | 390-458 | 390-459 | I00-I99 |
| Other natural cause | NA | NA | The rest of the codes | The rest of the codes | The rest of the codes |
| Unnatural death | NA | NA | 7959, 79621, 800-999 | 798, 800-999 | R95, R96, R98, V01-Y98 |
| The outcome of interest |  |  |  |  |  |
| Atrial fibrillation | NA | NA | 427.92 | 427D | I48 |
| Other variables |  |  |  |  |  |
| Maternal hypertension | NA | NA | 400-404, 63701, 63703, 63704, 63709, 63710 | 401-405, 642 | I10-I15, O10, O11, O13- O16 |
| Maternal diabetes | NA | NA | 250 | 250, 648A | E10-E14, O24 |
| Cardiovascular diseases in parents | NA | NA | 390-458 | 390-459 | I00-I99 |
| Psychiatric diseases in parents | NA | NA | 290-315 | 290-319 | F00-F99 |
| Cardiovascular diseases in the family | 400-468 | 400-468 | 390-458 | 390-459 | I00-I99 |
| Psychiatric diseases in the family | NA | NA | 290-315 | 290-319 | F00-F99 |
| Acute myocardial infarction in the offspring | NA | NA | 410 | 410 | I21, I22 |
| Heart failure in the offspring | NA | NA | 42700, 42710 | 428 | I11.0, I13.0, I13.2, I50 |
| Hypertension in the offspring | NA | NA | 400-404, 63701, 63703, 63704, 63709, 63710 | 401-405, 642 | I10-I15, O10, O11, O13- O16 |
| Diabetes in the offspring | NA | NA | 250 | 250, 648A | E10-E14, O24 |
| Psychiatric diseases in the offspring | NA | NA | 290-315 | 290-319 | F00-F99 |

*ICD* International Classification of Diseases, *NA* not available.

^a^In Denmark ICD-8 was used during 1970-1993, while ICD-10 during 1994-2018. In Sweden ICD-6 was used during 1952-57, ICD-7 during 1958-1968, ICD-8 during 1969-1986, ICD-9 during 1987-1996 9 and ICD-10 during 1997-2014.

**Table S3.** Adjusted incidence rate ratios for atrial fibrillation by death of a parent or a sibling among offsprings with data on specific covariates

| **Covariates adjusted for in addition to factors in the base model** |  | **Any loss in childhood** | | **Any loss in adulthood** | |
| --- | --- | --- | --- | --- | --- |
|  | **N^a^** | **Main model^b^** | **Main model^b^+ covariate** | **Main model^b^** | **Main model^b^+ covariate** |
|  |  | **IRR (95% CI)** | **IRR (95% CI)** | **IRR (95% CI)** | **IRR (95% CI)** |
| The offspring’s gestational age at birth | 5944770 | 1.24 (1.12-1.37) | 1.23 (1.12-1.36) | 1.26 (1.15-1.38) | 1.26 (1.14-1.38) |
| Maternal and paternal income at offspring’s birth | 6211716 | 1.26 (1.15-1.38) | 1.26 (1.15-1.38) | 1.25 (1.15-1.35) | 1.25 (1.16-1.36) |
| Maternal smoking in early pregnancy | 4222773 | 1.12 (0.90-1.39) | 1.12 (0.90-1.39) | 1.47 (1.09-2.00) | 1.46 (1.08-1.99) |
| Maternal BMI in early pregnancy | 2946995 | 1.45 (1.11-1.90) | 1.45 (1.11-1.89) | 1.46 (1.01-2.12) | 1.45 (1.00-2.11) |
| Maternal hypertension before offspring’s birth | 6096577 | 1.25 (1.14-1.37) | 1.25 (1.14-1.37) | 1.25 (1.14-1.36) | 1.25 (1.14-1.36) |
| Maternal diabetes before offspring’s birth | 6096577 | 1.25 (1.14-1.37) | 1.25 (1.14-1.37) | 1.25 (1.14-1.36) | 1.25 (1.14-1.36) |
| Parents’ CVD before offspring’s birth | 6096577 | 1.25 (1.14-1.37) | 1.25 (1.14-1.37) | 1.25 (1.14-1.36) | 1.25 (1.14-1.36) |
| Family history of CVD before offspring’s birth | 5076509 | 1.12 (0.98-1.28) | 1.11 (0.97-1.27) | 1.33 (1.18-1.48) | 1.32 (1.18-1.48) |
| Family history of psychiatric disorders before offspring’s birth | 4923155 | 1.17 (1.02-1.34) | 1.16 (1.01-1.33) | 1.25 (1.11-1.41) | 1.25 (1.11-1.40) |

*IRR* incidence rate ratio, *CI* confidence intervals, *BMI* body-mass index, *CVD* cardiovascular diseases.

^a^The number of offsprings without missing data on each covariate in the fully adjusted model.

^b^Adjusted for time since birth, calendar year, country, maternal age at offspring’s birth, and the parents’ country of origin, highest education and history of psychiatric disorders.

**Table S4.** Adjusted incidence rate ratios for atrial fibrillation according to bereavement in analyses considering potential mediators

| **Potential mediators** | **Exposure** | | | | | | | |
| --- | --- | --- | --- | --- | --- | --- | --- | --- |
|  | **Loss in childhood** | | | | **Loss in adulthood** | | | |
|  | **With potential mediator after loss** | | | | **With potential mediator after loss** | | | |
|  | **No** | | **Yes** | | **No** | | **Yes** | |
|  | **Events/**  **person-years** | **Adjusted IRR (95% CI)^a^** | **Events/**  **person-years** | **Adjusted IRR (95% CI)^a^** | **Events/**  **person-years** | **Adjusted IRR (95% CI)^a^** | **Events/**  **person-years** | **Adjusted IRR (95% CI)^a^** |
| Heart failure | 591/4401101 | 1.20 (1.10-1.30) | 24/8703 | 18.78 (12.57-28.07) | 898/2795881 | 1.18 (1.10-1.27) | 52/5460 | 32.39 (24.62-42.63) |
| AMI | 607/4402579 | 1.23 (1.13-1.34) | 8/7225 | 6.59 (3.29-13.18) | 935/2795475 | 1.23 (1.14-1.32) | 15/5866 | 8.03 (4.83-13.35) |
| Hypertension | 574/4291387 | 1.20 (1.10-1.31) | 41/118417 | 2.28 (1.67-3.10) | 892/2720118 | 1.20 (1.12-1.29) | 58/81224 | 2.53 (1.95-3.28) |
| Diabetes | 602/4331508 | 1.24 (1.14-1.35) | 13/78297 | 1.20 (0.69-2.06) | 929/2757738 | 1.23 (1.15-1.33) | 21/43603 | 1.71 (1.11-2.63) |
| Psychiatric disorders | 460/3394983 | 1.21 (1.10-1.33) | 155/1014822 | 1.34 (1.14-1.58) | 842/2486579 | 1.23 (1.14-1.33) | 108/314762 | 1.33 (1.10-1.62) |

*IRR* incidence rate ratio, *CI* confidence intervals, *AMI* acute myocardial infarction.

^a^Adjusted for time since birth, calendar year, country, maternal age at offspring’s birth, and the parents’ country of origin, highest education and history of psychiatric disorders.

**Table S5.** Adjusted incidence rate ratios for the association between death of a parent or a sibling in childhood and adulthood and atrial fibrillation in stratified analyses

|  | **Events/person-years** | **Adjusted IRR (95% CI)^a^** |
| --- | --- | --- |
| Sex of the child |  |  |
| Men |  |  |
| Unexposed | 5133/65632507 | 1.00 |
| Any loss in childhood | 419/2266702 | 1.17 (1.06-1.29) |
| Any loss in adulthood | 676/1446546 | 1.20 (1.11-1.31) |
| Women |  |  |
| Unexposed | 2025/62264905 | 1.00 |
| Any loss in childhood | 196/2142927 | 1.43 (1.23-1.66) |
| Any loss in adulthood | 274/1354769 | 1.35 (1.18-1.54) |
| Country |  |  |
| Denmark |  |  |
| Unexposed | 3528/56813010 | 1.00 |
| Any loss in childhood | 344/2199674 | 1.21 (1.08-1.35) |
| Any loss in adulthood | 577/1599288 | 1.19 (1.08-1.31) |
| Sweden |  |  |
| Unexposed | 3630/71088111 | 1.00 |
| Any loss in childhood | 271/2210131 | 1.27 (1.12-1.44) |
| Any loss in adulthood | 373/1202054 | 1.32 (1.19-1.48) |
| Highest educational level of the parents |  |  |
| 0-9 years |  |  |
| Unexposed | 784/10629665 | 1.00 |
| Any loss in childhood | 151/896179 | 1.26 (1.05-1.51) |
| Any loss in adulthood | 218/555634 | 1.30 (1.10-1.52) |
| 10-14 years |  |  |
| Unexposed | 4315/75269033 | 1.00 |
| Any loss in childhood | 327/2522583 | 1.19 (1.06-1.33) |
| Any loss in adulthood | 547/1641341 | 1.25 (1.14-1.37) |
| ≥15 years |  |  |
| Unexposed | 2050/41860340 | 1.00 |
| Any loss in childhood | 136/967382 | 1.37 (1.15-1.64) |
| Any loss in adulthood | 184/600593 | 1.16 (0.99-1.46) |

*IRR* incidence rate ratio, *CI* confidence intervals.

^a^Adjusted for time since birth, calendar year, maternal age at offspring’s birth, country, and the parents’ country of origin, highest education and history of psychiatric disorders.
